# Patient Care Technician Staffing in US Hemodialysis Facilities: An Ecological Study

**DOI:** 10.1016/j.xkme.2023.100782

**Published:** 2023-12-15

**Authors:** Laura C. Plantinga, Megan Urbanski, Courtney Hoge, Fran Rickenbach, Clarica Douglas-Ajayi, Jennifer Craft Morgan, Alexis A. Bender, Bernard G. Jaar

**Affiliations:** 1 Divisions of Rheumatology and Nephrology, University of California, San Francisco, San Francisco, California; 2 Departments of Surgery, Emory University, Atlanta, Georgia; 3 Medicine, Emory University, Atlanta, Georgia; 4 National Association of Nephrology Technicians/Technologists, Dayton, Ohio; 5 Gerontology Institute, Georgia State University, Atlanta, Georgia; 6 Departments of Medicine and Epidemiology and Welch Center for Prevention, Epidemiology, and Clinical Research, Johns Hopkins University, Baltimore, Maryland

**Keywords:** Dialysis facility, dialysis technician, in-center hemodialysis, patient care technician, staffing, United States

## Abstract

**Rationale & Objective:**

Technicians caring for patients receiving dialysis play a critical, frontline role in the care of patients receiving dialysis in the United States. We sought to provide a comprehensive description and identify correlates of US in-center hemodialysis facility patient care technician staffing patterns.

**Study Design:**

This was an ecological study.

**Setting & Participants:**

US facilities providing hemodialysis and reporting patient care technician staffing, identified using the US Renal Data System.

**Exposures:**

Geography, year, and facility characteristics, including aggregated patient characteristics.

**Outcomes:**

The study outcome was facility-reported patient-to-patient care technician ratio.

**Analytical Approach:**

We examined patient-to-patient care technician ratios by US state and over time and also estimated the differences in patient-to-patient care technician ratios associated with facility characteristics, using robust regression with adjustment for facility-level covariates.

**Results:**

The median patient-to-patient care technician ratio among 6,862 US facilities in 2019 was 9.9 (25^th^-75^th^ percentiles, 8.2-12.0). Median 2019 patient-to-patient care technician ratios varied substantially by US state and region. There was an overall decline (from 10.6 to 9.9) in median patient-to-patient care technician ratios from 2004 to 2019, whereas the percentage of positions that were unfilled increased (from 2.8% to 3.5%). After adjustment, large dialysis organization status (β, −0.42; 95% CI, −0.61 to −0.23) and larger facility size (β, −0.51; 95% CI, −0.68 to −0.33) were associated with lower patient-to-patient care technician ratios. Higher patient-to-registered nurse (β, 0.80; 95% CI, 0.65-0.94) and patient-to-social worker (β, 0.53; 95% CI, 0.37-0.70) ratios, presence of licensed vocational nurses or licensed practical nurses at the clinic (β, 0.83; 95% CI, 0.53-1.12), and location in a poverty area (β, 0.29; 95% CI, 0.13-0.44) were all associated with higher patient-to-patient care technician ratios. Aggregated patient characteristics of patients treated at the facilities were generally not associated with patient-to-patient care technician ratio after adjustment.

**Limitations:**

Limited causal inference and potential shifts in staffing after 2019.

**Conclusions:**

US dialysis facilities vary considerably in their patient care technician staffing by geography, over time, and by various facility characteristics. Further investigation of US patient care technician staffing is warranted and could lead to better, more stable dialysis staffing, improved staff and patient satisfaction, and higher quality of care.

## Plain-Language Summary

In the United States, patient care technicians play an important role in hemodialysis care. Although ongoing staffing shortages and turnover among other hemodialysis care providers have been described, little is known about US patient care technician staffing. Examining national data reported by dialysis facilities, we found variability in patient care technician staffing by geography, over time (with fewer patients per patient care technician in more recent years), and by various facility characteristics. This information can be used to target staff recruitment and retention interventions at facilities where patient care technician staffing may be more challenging.

Editorial, 100795

In the United States, in-center hemodialysis (HD) care is delivered by a Centers for Medicare & Medicaid Services (CMS)-mandated interdisciplinary team (physicians, nurses, social workers, dietitians, and technicians caring for patients receiving dialysis), designed to meet the complex needs of patients receiving HD.^1^ Technicians caring for patients receiving dialysis play a critical, frontline role in the care of patients receiving dialysis in the United States. Primarily under the supervision of registered nurses, they manage many of the technical aspects of dialysis, including the operation and disinfection of dialysis machines, needle insertion, and collection and documentation of patient vital signs. Additionally, as the staff members who have the most face-to-face time with patients receiving dialysis, patient care technicians may play an important role in providing education to patients and their caregivers and serve as liaisons between patients and other staff at the clinic.

However, the effectiveness of the patient care technician’s role in delivering high-quality dialysis care may be undermined by high levels of turnover at US dialysis facilities. In a recent survey of technicians caring for US patients receiving dialysis, only about half intended to continue working as patient care technicians and, of these, only 69% intended to continue working at the same facility.^2^ Consistently high levels of turnover can lead to increased burden on remaining patient care technicians, as well as other dialysis staff—particularly, nurses—straining an already overburdened and burned-out dialysis workforce.^3^, ^4^, ^5^

Although the number of technicians caring for patients receiving dialysis continues to grow with the patient population (>47,000 technicians caring for patients receiving dialysis in 2018^1^), and several states have instituted dialysis staffing mandates (Table S1),^6^^,^^7^ little is known about how patient care technician staffing patterns differ across US dialysis facilities. Here, we leverage national data to provide a comprehensive description of dialysis patient care technician staffing patterns and to identify facility characteristics that are associated with differences in staffing of technicians caring for patients receiving dialysis.

## Methods

### Study Population and Data Sources

We obtained CMS facility (CMS End-Stage Renal Disease [ESRD] Annual Facility Survey [AFS; CMS-2744] and CMS Dialysis Facility Compare) and patient (Core and ESRD Medical Evidence Report [CMS-2728]) data from the US Renal Data System (USRDS).^8^ The Emory University and University of California, San Francisco Institutional Review Boards declared the study exempt, with a waiver of informed consent because of inability to identify participants. AFS data (annual survey periods, from January 1 to December 31) were available through 2019.

**Figure 1:**
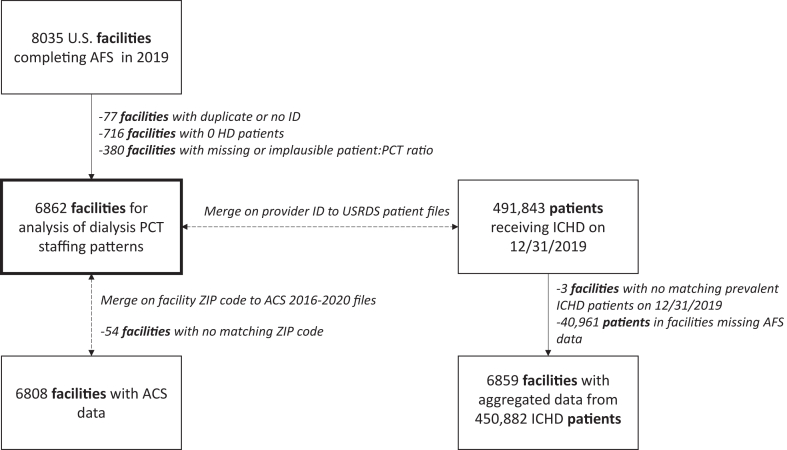
Selection of facility-level analytic data from the US Renal Data System for analysis. ACS, American Community Survey; AFS, Annual Facility Survey; HD, hemodialysis; ICHD, in-center hemodialysis; PCT, patient care technician.

For this ecological study, we primarily examined patterns of patient-to-patient care technician ratios in 2019. Of the 8,035 US dialysis facilities with AFS data in 2019, we excluded facilities that were duplicates (n = 77), reported 0 in-center patients receiving HD (n = 716), or had missing or implausible patient-to-patient care technician ratio values (<1 or >96, considered the maximum for 4 shifts per day, 6 days per week, 4 patients per shift; n = 380), leaving 6,862 facilities for primary analysis (Fig 1). For analyses examining patterns over time, the same exclusion criteria were applied for each survey year. USRDS patient data were linked by the USRDS provider ID on December 31, 2019. There were 450,882 point prevalent in-center patients receiving HD from 6,859 facilities who had linked AFS data (Fig 1). Additionally, ZIP code tabulation area-level American Community Survey data from 2016 to 2020^9^ were linked by facility ZIP code, with n = 6,808 matches (Fig 1).

### Study Variables

Patient-to-patient care technician ratio. Census of patients receiving HD at the facility (reported number of patients who, at the end of the survey period, were receiving staff-assisted hemodialysis or performing outpatient self-hemodialysis) and number of technicians caring for patients receiving dialysis were obtained from the USRDS facility data. The number of technician full-time equivalents (FTEs) caring for patients receiving dialysis were estimated using: 1 × (number of full-time technicians caring for patients receiving dialysis at the end of the survey period) + 0.5 × (number of part-time technicians caring for patients receiving dialysis at the end of the survey period). The patient-to-patient care technician ratio in each year was then defined as: (census of patients receiving HD on December 31 of the survey year) / (number of technician FTEs caring for patients receiving dialysis on December 31 of the survey year).

Facility variables. Facility variables, extracted from the provider-reported AFS, included ownership (for-profit or not-for-profit), type (freestanding or hospital-based), patient-to-staff ratios (for registered nurses and social workers), presence of an advanced practice provider or licensed practical or vocational nurse (LPN or LVN), US state or territory, and number of HD treatments provided on an outpatient basis in the prior year. Large dialysis organization (LDO) status (≥200 dialysis facilities) was determined by the facility chain. Facility states were divided into US regions (with US territories included in the West because of small numbers), as well as CMS-defined ESRD Networks (with northern and southern California defined by facility ZIP code). HD station-to-patient care technician ratios were estimated as (number of HD stations reported during the survey period) / (technician FTEs caring for patients receiving dialysis). Treatment-to-patient care technician ratios were estimated as (number of HD treatments provided during the survey period) / (technician FTEs caring for patients receiving dialysis).

Aggregated patient-level variables. Estimated patient age (reported incident patient age + number of years since dialysis start on December 31, 2019); provider-reported sex, race, and ethnicity; receipt of predialysis nephrology care; diabetes at dialysis start; functional impairment (inability to ambulate or transfer, needing assistance with activities of daily living, and/or institutionalization) at dialysis start; and permanent vascular access (arteriovenous graft or fistula in place or maturing) at dialysis start were all obtained from the provider-reported CMS 2728 data for patients receiving in-center hemodialysis on December 31, 2019. Waitlisted status was obtained from United Network for Organ Sharing data that were included in the USRDS. All patient-level variables were aggregated to the facility level.

Facility neighborhood variables. Percentages with highest level of education completed at high school graduate or equivalent and college graduate level and percentage living below poverty level at the ZIP code tabulation area level were obtained from American Community Survey data.^9^

### Statistical Analysis

Characteristics of the included facilities in the most recent survey year (2019) were summarized as percentages, means, or medians. Dialysis patient care technician staffing ratios in 2019 were summarized overall and by facility, neighborhood, and facility-aggregated patient characteristics, using Wilcoxon rank sum tests. Patient care technician staffing ratios were also summarized by survey year and at the US state and ESRD Network levels. Additionally, the associations of 2019 patient care technician staffing ratios with selected facility, neighborhood, and facility-aggregated patient characteristics were assessed using robust regression, which eliminates gross outliers by Cook’s distance calculations and estimates parameters by Huber and then biweight iterations,^10^ with complete case analysis of nonmissing data. We also performed sensitivity analyses by examining the HD station-to-patient care technician ratio as an outcome, to assess the robustness of the associations to a different operationalization of the staffing ratio and including only facilities that had been open for at least 5 years (2015-2019) with ≤10% (n = 161) and ≤5% (n = 480) variation in patient census, to assess the robustness of the associations of patient-to-patient care technician ratio with characteristics among established in-center HD facilities. All analyses were performed with Stata version 18.0.

## Results

### Characteristics of Facilities

Table 1 shows the characteristics for the 6,862 included US dialysis facilities in 2019. Overall, 73.3% of facilities represented LDOs, and 89.6% operated on a for-profit basis. Among the non-LDO facilities, 62.6% were for-profit. Most (95.8%) were freestanding, and the facilities had a median of 17 in-center HD stations. More than two-thirds of facilities were in the US South and Midwest regions (Table 1); >20% were in ESRD Network 6 (Georgia, North Carolina, South Carolina) or Network 14 (Texas; Table S2). The median patient-to-patient care technician ratio in 2019 was 9.9, with a median of 3.0 stations per patient care technician and 1,456.1 annual treatments per patient care technician (Table 1). Facilities had medians of 15 patients per registered nurse and 70 patients per social worker. Advanced practice providers and LVNs or LPNs were uncommon, with 4.5% and 6.1% of facilities reporting these workers.

**Table 1:** Characteristics of 6,862 US Facilities Offering In-center Hemodialysis and Employing Patient Care Technicians as of December 31, 2019

Characteristic	N	Value
**General facility characteristics**		
Ownership, n (%)	6,862	
For-profit		6,147 (89.6%)
Not-for-profit		715 (10.4%)
Large dialysis organization, n (%)	6,862	
Yes		5,031 (73.3%)
No		1,831 (26.7%)
Type, n (%)	6,862	
Hospital-based		288 (4.2%)
Freestanding		6,574 (95.8%)
Total number of stations, median (IQR)	6,856	17.0 (13.0-24.0)
Region of facility	6,862	
Northeast		976 (14.2%)
South		3,167 (46.2%)
Midwest		1,489 (21.7%)
West or US Territories		1,230 (17.9)
**Facility staffing**		
Patient-to-PCT ratio, median (IQR)	6,862	9.9 (8.2-12.0)
Station-to-PCT ratio, median (IQR)^a^	6,818	3.0 (2.3-4.3)
Treatment-to-PCT ratio, median (IQR)^a^	6,862	1,456.1 (1,191.6-1,777.8)
% of PCT positions open, mean (SD)	6,862	3.5% (9.1%)
Patient-to-RN ratio, median (IQR)	6,857	15.0 (11.0-19.8)
Patient-to-SW ratio, median (IQR)	6,742	70.0 (48.0-93.0)
APP present, n (%)	6,862	
Yes		306 (4.5%)
No		6,556 (95.5%)
LVN or LPN present, n (%)	6,862	
Yes		418 (6.1%)
No		6,444 (93.9%)
**Facility-aggregated patient characteristics**^b^		
Median prevalent patient count (IQR)	6,859	58.0 (37.0-86.0)
Mean % aged ≥65 y (SD)^c^	6,859	48.6% (12.8%)
Mean % male (SD)	6,859	57.5% (9.1%)
Mean % African American (SD)	6,859	33.3% (29.2%)
Mean % White (SD)	6,859	60.2% (28.7%)
Mean % with diabetes at dialysis start (SD)	6,859	58.1% (11.5%)
Mean % with functional impairment at dialysis start (SD)	6,859	12.8% (9.7%)
Mean % with known predialysis nephrology care (SD)	6,858	66.6% (16.2%)
Mean % with permanent vascular access in place or maturing at dialysis start (SD)	6,858	42.9% (14.8%)
Mean % on transplant waitlist (SD)	6,859	8.1% (6.7%)
**Facility neighborhood socioeconomic characteristics**^d^		
Highest level of education completed		
Mean % high school degree or equivalent (SD)	6,798	27.8% (9.2%)
Mean % bachelor’s degree (SD)	6,309	18.8% (8.5%)
Mean % below poverty level (SD)	6,791	25.2% (10.0%)

Abbreviations: APP, advanced practice provider; HD, hemodialysis; IQR, interquartile range (25^th^-75^th^ percentiles); LVN or LPN, licensed vocational nurse or licensed practical nurse; PCT, patient care technician, RN, registered nurse; SD, standard deviation; SW, social worker. Minimum and maximum values for patient-to-PCT ratios were 1 and 96, respectively.

a Treatment-to-PCT ratios were estimated as (number of HD treatments provided during the survey period) / (dialysis PCT full-time equivalents).

b Aggregated over patients receiving in-center HD on December 31, 2019. N = 450,882 patients in 4,859 facilities.

c Estimated on December 31, 2019 based on incident age and dialysis start date.

d Based on the facility ZIP code tabulation area; data from the 2016-2020 American Community Survey, which was available for 6,808 facilities.

The facilities had a median of 58 prevalent patients on December 31, 2019. Table 1 lists patient characteristics aggregated to the facility level, including mean percentages of patients aged ≥65 years (48.6%), patients who were male (57.5%), and patients who were African American (33.3%). A mean of 58.1% of patients had diabetes, and 12.8% had functional impairment at dialysis start (Table 1). The highest levels of education completed for 27.8% and 18.8% of individuals within facility neighborhoods (ZIP code tabulation areas) were high school graduate and bachelor’s degree, respectively, whereas a mean of 25.2% of households within facility ZIP code tabulation areas lived below the poverty level (Table 1).

### Dialysis Patient Care Technician Staffing by Geography and Over Time

**Figure 2:**
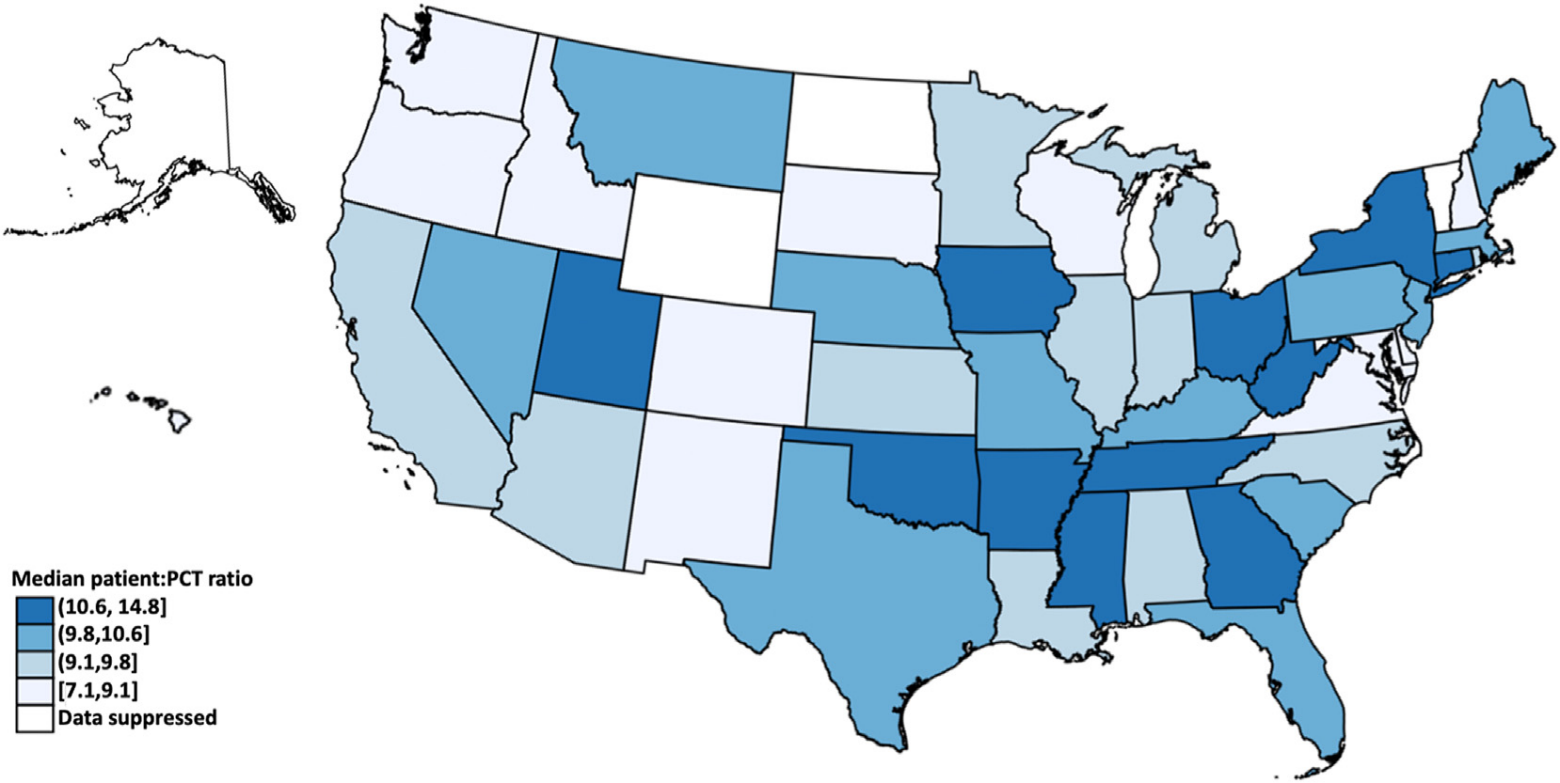
Median US dialysis facility patient-to-patient care technician ratios in 2019, by state. Data from states with 10 or fewer facilities were suppressed. PCT, patient care technician.

A map of median patient-to-patient care technician ratios at the US state level is shown in Fig 2. The highest state-level ratios (≥11) were seen in New York, Connecticut, Ohio, West Virginia, Tennessee, Georgia, Mississippi, Arkansas, Oklahoma, Iowa, North Dakota, and Utah. Similarly, the highest median station-to-patient care technician ratios (≥3.8) were seen in Connecticut, Ohio, West Virginia, Tennessee, Kentucky, Georgia, Arkansas, Oklahoma, Iowa, North Dakota, and Utah (Fig S1). Table S2 shows that ESRD Networks 2 (New York; 12.1), 8 (Alabama, Mississippi, Tennessee; 10.7), 1 (New England; 10.5), 3 (New Jersey, Puerto Rico, US Virgin Islands; 10.5), and 14 (Texas; 10.5) had the highest patient-to-patient care technician ratios, whereas Networks 4 (Iowa, Kansas, Missouri, Nebraska; 4.0), 8 (Alabama, Mississippi, Tennessee; 3.8), 9 (Indiana, Kentucky, Ohio; 3.7), and 13 (Arkansas, Louisiana, Oklahoma; 3.7) had the highest station-to-patient care technician ratios. Network 16 (Pacific Northwest and Alaska) had the lowest median patient-to-patient care technician ratio (8.0) and station-to-patient care technician ratio (2.0) in 2019.

**Figure 3:**
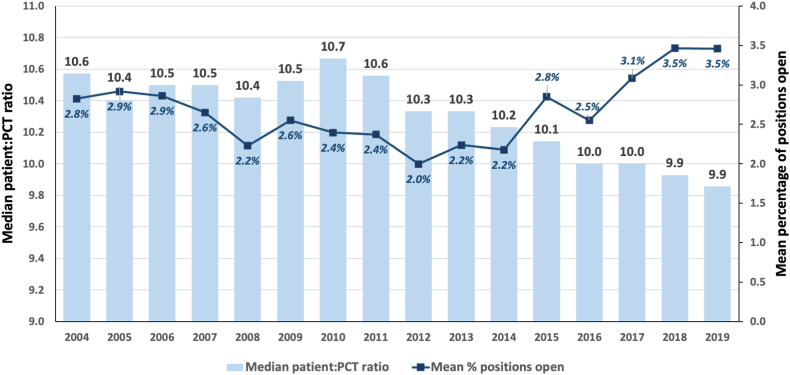
Median facility patient-to-patient care technician ratios (*bars*) and mean percentage of dialysis patient care technician positions reported as open (*squares*) at US dialysis facilities, by year. *P* < 0.001 across year for both. PCT, patient care technician.

Fig 3 shows that the median patient-to-patient care technician ratio among US facilities offering in-center HD declined from 2004 (10.6) to 2019 (9.9; *P* < 0.001). In 2016-2019, all median patient-to-patient care technician ratios were ≤10. In contrast, the mean percentage of patient care technician positions that were reported as open or unfilled as of the end of the survey year by US facilities increased from 2.8% in 2004 to 3.5% in 2019 (Fig 3). The median station-to-patient care technician ratio remained consistent across this same time period, with only a slight increase in 2010-2013 (Fig S2).

### Association of Facility Characteristics with Dialysis Patient Care Technician Staffing Ratios

Median 2019 patient-to-patient care technician ratios were lower among LDO versus non-LDO facilities (9.6 vs 10.0, *P* < 0.001) and facilities with higher versus lower percentages of patients with permanent vascular access in place or maturing at dialysis start (9.8 vs 10.0, *P* = 0.007). In contrast, patient-to-patient care technician ratios were higher among facilities with higher versus lower patient-to-registered nurse ratios (10.3 vs 9.4, *P* < 0.001) and patient-to-social worker ratios (10.2 vs 9.4, *P* < 0.001); facilities with LVNs or LPNs present versus absent (11.0 vs 9.8, *P* < 0.001); facilities with higher versus lower percentages of patients with functional impairment (10.0 vs 9.8, *P* < 0.001); and facilities in poverty versus nonpoverty areas (>20% poverty; 10.0 vs 9.6, *P* < 0.001; Table 2). Correlations of patient-to-patient care technician ratios with selected continuous factors were generally weak (Fig S3).

**Table 2:** Association of Selected Facility Characteristics With 2019 US Facility Patient-to-Patient Care Technician Ratios

Characteristic	Median (IQR) Patient-to-PCT Ratio	Difference in Patient-to-PCT Ratio [β (95% CI)^a^]		
		Crude	Model 1^b^	Model 2^b^
**Ownership**				
For-profit	9.9 (8.3-12.0)	1.00 (ref.)	1.00 (ref.)	1.00 (ref.)
Not-for-profit	9.7 (7.6-12.6)	−0.55 (−0.79 to −0.32)	−0.26 (−0.55 to 0.02)	−0.22 (−0.50 to 0.07)
P^c^	0.08			
**Large dialysis organization**				
Yes	10.0 (8.3-12.0)	1.00 (ref.)	1.00 (ref.)	1.00 (ref.)
No	9.6 (7.8-12.1)	−0.45 (−0.61 to −0.29)	−0.34 (−0.53 to −0.15)	−0.42 (−0.61 to −0.23)
P^c^	<0.001			
**Type**				
Freestanding	9.9 (8.3-12.0)	1.00 (ref.)	1.00 (ref.)	1.00 (ref.)
Hospital-based	9.3 (6.4-15.0)	−1.43 (−1.79 to −1.07)	−1.15 (−1.58 to −0.72)	−0.92 (−1.36 to −0.49)
P^c^	0.08			
**Number of stations**				
<18	9.8 (8.0-12.4)	1.00 (ref.)	1.00 (ref.)	1.00 (ref.)
≥18	9.9 (8.4-11.8)	0.20 (0.06-0.35)	−0.49 (−0.67 to −0.32)	−0.51 (−0.68 to −0.33)
P^c^	0.3			
**Patient-to-registered nurse ratio**				
≤15	9.4 (7.5-11.8)	1.00 (ref.)	1.00 (ref.)	1.00 (ref.)
>15	10.3 (8.8-12.4)	1.04 (0.90-1.18)	0.81 (0.66-0.95)	0.80 (0.65-0.94)
P^c^	<0.001			
**Patient-to-social worker ratio**				
≤70	9.4 (7.7-11.8)	1.00 (ref.)	1.00 (ref.)	1.00 (ref.)
>70	10.2 (8.7-12.3)	0.88 (0.74-1.03)	0.54 (0.37-0.71)	0.53 (0.37-0.70)
P^c^	<0.001			
**Advanced practice provider present**				
No	9.9 (8.2-12.0)	1.00 (ref.)	1.00 (ref.)	1.00 (ref.)
Yes	10.0 (8.0-12.0)	0.05 (−0.30 to 0.40)	0.12 (−0.22 to 0.46)	0.15 (−0.20 to 0.49)
P^c^	0.8			
**LVN or LPN present**				
No	9.8 (8.1-12.0)	1.00 (ref.)	1.00 (ref.)	1.00 (ref.)
Yes	11.0 (8.8-14.4)	1.02 (0.72-1.32)	0.87 (0.57-1.16)	0.83 (0.53-1.12)
P^c^	<0.001			
**Percentage of patients who are African American**				
≤25%	9.8 (8.0-12.0)	1.00 (ref.)	1.00 (ref.)	1.00 (ref.)
>25%	10.0 (8.3-12.0)	0.16 (0.01-0.30)	0.08 (−0.06 to 0.22)	0.09 (−0.17 to 0.35)
P^c^	0.2			
**Percentage of patients with permanent vascular access in place or maturing at dialysis start**				
≤40%	10.0 (8.3-12.2)	1.00 (ref.)	1.00 (ref.)	1.00 (ref.)
>40%	9.8 (8.0-12.0)	−0.17 (−0.32 to −0.03)	−0.14 (−0.28 to 0.00)	−0.15 (−0.38 to 0.08)
P^c^	0.007			
**Percentage of patients on kidney transplant waitlist**				
≤7%	10.0 (8.2-12.4)	1.00 (ref.)	1.00 (ref.)	1.00 (ref.)
>7%	9.8 (8.2-12.0)	−0.14 (−0.28 to 0.01)	−0.26 (−0.41 to −0.12)	−0.22 (−0.36 to −0.07)
P^c^	0.01			
**Percentage of patients with functional impairment**				
≤10%	9.8 (8.0-12.0)	1.00 (ref.)	1.00 (ref.)	1.00 (ref.)
>10%	10.0 (8.3-12.3)	0.22 (0.07-0.36)	0.27 (0.13-0.41)	0.24 (0.04-0.43)
P^c^	<0.001			
**Facility in poverty area**^d^				
No	9.6 (8.0-11.8)	1.00 (ref.)	1.00 (ref.)	1.00 (ref.)
Yes	10.0 (8.3-12.3)	0.38 (0.22-0.53)	0.31 (0.16-0.46)	0.29 (0.13-0.44)
P^c^	<0.001			

*Note:* N = 6,858 (excluding observations with missing data on covariates).

Abbreviations: CI, confidence interval; IQR, interquartile range (25^th^-75^th^ percentiles); LVN or LPN, licensed vocational nurse or licensed practical nurse; PCT, patient care technician; ref., reference.

a From robust regression.^10^

b Model 1 adjusted for facility characteristics: ownership (profit or not-for-profit), large dialysis organization (yes vs no), number of prevalent in-center hemodialysis patients. Model 2 adjusted for variables in Model 1 plus patient and neighborhood characteristics: percentage of patients who were aged ≥65, percentage of patients who were African American, percentage of patients with diabetes, percentage of patients with functional impairment, percentage of patients with a permanent vascular access in place at dialysis start, and percentage of patients with known predialysis care.

c By Wilcoxon rank sum test.

d N = 6,787. Poverty area defined as a ZIP code tabulation area with ≥20% of households living below the poverty level.

In adjusted models, LDO versus non-LDO status remained associated with 0.4 fewer patients per patient care technician (Table 2). Similarly, being hospital-based and having more stations were associated with 0.9 and 0.5 fewer patients per patient care technician. Higher staff ratios were associated with more patients per patient care technician. Specifically, having a higher patient-to-registered nurse ratio was associated with 0.8 more patients per patient care technician, having a higher patient-to-social worker ratio was associated with 0.5 more patients per patient care technician, and having LVNs or LPNs present was associated with 0.8 more patients per patient care technician (Table 2). However, advanced practice provider presence was not associated with patient-to-patient care technician ratio. Although having more patients with functional impairment was associated with higher patient-to-patient care technician ratios (β = 0.2), percentages of patients who were African American and who had a permanent vascular access in place were not associated with patient-to-patient care technician ratio. Facilities with higher-than-median versus lower-than-median percentages of patients on the waitlist had lower patient-to-patient care technician ratios after adjustment (β = −0.2). Facility location in a poverty area (≥20% of households living below the poverty level) was associated with 0.3 more patients per patient care technician (Table 2).

In sensitivity analyses, not-for-profit status, larger size, presence of LVNs or LPNs, and location in a poverty area were associated with higher HD station-to-patient care technician ratios, whereas being hospital-based and having a higher patient-to-social worker ratios were associated with lower station-to-patient care technician ratios (Table S3). Facilities with ≤10% and ≤5% variation in patient census in 2015-2019 had lower overall patient-to-patient care technician ratios (median [interquartile range] of 9.5 [7.5-12.0] and 8.0 [5.9-10.6], respectively). When analyses were restricted to facilities with ≤10% variation, associations of higher patient-to-registered nurse, higher patient-to-social worker ratios, presence of LVNs or LPNs, and higher percentages of patients with functional impairment with higher patient-to-patient care technician ratios were consistent with the main analyses (Table S4). When analyses were restricted to those with ≤5% variation, higher patient-to-registered nurse and higher patient-to-social worker ratios were more strongly associated with higher patient-to-patient care technician ratios, compared with the main analyses, whereas presence of LVNs or LPNs was not associated with patient-to-patient care technician after adjustment (Table S5).

## Discussion

We found that, in 2019, US in-center HD facilities had an overall median of 9.9 patients per dialysis patient care technician FTE, with wide variability: one-quarter of facilities had patient-to-patient care technician ratios of ≤8.2 and another quarter had patient-to-patient care technician ratios of ≥12.0. Facilities also reported a median of 3.0 HD stations per patient care technician and nearly 1,500 HD treatments per patient care technician in 2019. Patient-to-patient care technician ratios varied substantially by US state and ESRD Network in 2019, whereas there was an overall decline (from 10.6 to 9.9) in median patient-to-patient care technician ratios from 2004 to 2019. Several facility characteristics were associated with higher patient-to-patient care technician ratios, including non-LDO status, larger facility size, higher patient-to-registered nurse and patient-to-social worker ratios, presence of LVNs or LPNs at the clinic, and location in a poverty area. Characteristics of patients treated at the facilities were generally not associated with patient-to-patient care technician ratio after adjustment.

Interestingly, the geographic patterns we observed did not necessarily align with existing state mandates for patient care technician staffing.^6^^,^^7^ Among the states with staffing mandates, only Maryland and Oregon had low patient-to-patient care technician ratios in 2019; Massachusetts, Nevada, South Carolina, and Texas had high ratios, whereas Georgia and Utah had very high patient-to-patient care technician ratios. In several cases, the median station-to-patient care technician ratios were low, potentially suggesting high overall patient loads but fewer patients per shift (ie, more shifts per patient care technician). However, Georgia, Texas, and Utah also had high median station-to-patient care technician ratios relative to other states, which may suggest unique regional problems with patient care technician staffing and/or retention. There are also substantial state-level differences in types of facilities (for-profit vs not-for-profit, LDO vs other) offering employment, patient care technician certification programs, on-the-job clinical experience requirements, and other state regulations governing allowable patient care technician tasks,^11^^,^^12^ all of which may affect the ability of facilities in certain states or regions to attract and retain patient care technicians. The effect of the coronavirus disease 2019 (COVID-19) pandemic on staffing may have also been experienced differentially by state.

Although the median patient-to-patient care technician ratio did decrease from 2004 to 2019, the mean percentage of patient care technician positions that were reported as open increased (from 2.8% to 3.5%). This may reflect increasing turnover (patient care technicians moving between facilities, switching from dialysis patient care technician to another job, or retiring). This pattern may also reflect increased hiring efforts by facilities to keep up with the rapidly increasing US in-center population of patients receiving HD (from 301,640 in 2004 to 492,987 prevalent patients in 2019, a 63% increase^8^), respond to staffing mandates,^6^^,^^7^ and/or meet increasing quality-of-care payment incentives^1^ over the same time period. Additionally, median station-to-patient care technician ratios remained fairly consistent from 2004 to 2019. This pattern, contrasting with the decreasing patient-to-patient care technician ratios, might reflect fewer shifts over time (ie, if facilities are eliminating less desirable shifts because of the inability to staff them), more patient care technicians being hired (as mentioned above), and/or differences in US facilities over time (if those that closed vs opened during this time period had different staffing patterns). This observation might also reflect that patient care technicians were treating fewer patients in a single facility overall but a similar number of patients (at a fixed number of stations) per shift by 2019. The effect of the pandemic on this pattern over time remains unknown, but in our recent postpandemic survey of technicians caring for patients receiving dialysis, we found that nearly one-quarter reported working at multiple facilities^2^; it is a limitation of our data that individual patient care technicians cannot be tracked across facilities. Thus, these numbers reflect only the facility-level burden per patient care technician FTE and not necessarily burdens experienced by individual patient care technicians.

We found that several facility characteristics were associated with patient care technician staffing. Although we found that LDO status was associated with higher patient-to-patient care technician ratios, there was no difference by this status in HD station-to-patient care technician ratios, which may reflect the ability of LDOs to maintain both larger facility sizes and greater numbers of shifts. However, larger facility size was associated with higher HD station-to-patient care technician ratios. Higher patient-to-registered nurse and patient-to-social worker ratios were associated with higher patient-to-patient care technician ratios, which suggests that staffing choices and challenges may be similar across dialysis care team roles within facilities. Although there is little direct evidence of staffing challenges being consistent across roles within dialysis facilities, shortages in other dialysis care providers overall (particularly nurses^13^^,^^14^ and with the COVID-19 pandemic^14^^,^^15^) have been well-documented. These associations were not seen with HD station-to-patient care technician ratio. There was no difference in either ratio by whether there were advanced practice providers present. However, the presence of LVNs or LPNs was associated with both higher patient-to-patient care technician and HD station-to-patient care technician ratios. LVNs or LPNs were uncommon among facilities reporting in 2019 (∼6%) but, when present, they may perform some of the tasks that would normally be delegated to patient care technicians, allowing patient care technicians to take on a higher patient load.^16^ Another possibility is that the presence of LVNs or LPNs reflects a facility having difficulty hiring patient care technicians, registered nurses, or both. Facility location in a poverty area was also associated with higher patient care technician staffing ratios; the potential added difficulty of hiring individuals in these areas—because of adverse payer mix limiting budgets for staff and/or other factors—may partially explain these associations. Many of the observed associations were stronger among more established facilities (ie, with only small changes in census over 5 years), suggesting that variability among facilities that are still growing may affect observed patient care technician staffing patterns and associations.

Our study provides several testable hypotheses for observational studies and potential targets for intervention studies aimed at increasing staff retention and/or improved patient care. However, one major limitation of our study is that the patient care technician staffing pattern for optimal levels of retention and quality of delivered care at the dialysis facility remains unknown. Future studies examining patient care technician staffing that include quality data, such as those from the ESRD QIP,^12^ are needed to assess staffing levels in context of the care provided by the facility. A definition of adequate patient care technician staffing is essential for studies assessing outcomes of interventions aimed at staff retention. Such a definition is also needed to determine the level of concern we should have over staffing patterns, although levels of burnout and turnover intention and reports of understaffing among technicians caring for patients receiving dialysis in our recent postpandemic surveys^2^^,^^17^ suggest that current patient care technician staffing is likely inadequate and that its possible effects on the well-being of dialysis staff and the quality of overall patient care should be examined more closely.

There are other limitations not noted above that deserve mention. First, there is a possibility of misclassification: the data from the AFS are self-reported by facilities and not verified by outside sources, and they only capture staffing at the end of the year rather than over the entire year. Additionally, another limitation of our study was our inability to estimate the number of patients seen by a patient care technician during a single shift because of lack of information on the number of shifts available (although our sensitivity analyses examining HD station-to-patient care technician ratios at least partially address this issue). Several of our assumptions, including that part-time patient care technicians contributed 0.5 FTE on average and that characteristics at the end of the survey period reflect the patterns seen throughout the year, may have introduced some degree of misclassification, which we would expect to be nondifferential. Additionally, only the total number of patients receiving HD (in-center and home HD combined) is collected on the AFS, but this was not likely to have a substantial impact on the results: as of 2019, only 1.8% of prevalent patients were receiving home HD.^1^ Second, the amount and type of training for patient care technicians at dialysis facilities is not reported on the AFS. Third, our data did not include 2020-2022, when there were likely substantial changes in staffing patterns (turnover and staffing shortages) with the onset of the COVID-19 pandemic.^5^ These results do provide information on the existing patterns of patient care technician staffing before the likely negative influence of the pandemic, which is still important for projecting the future of dialysis workforce staffing. Finally, this study is cross-sectional and ecological; thus, causal inference is limited.

Despite these limitations, this ecological study provides an important, comprehensive description of national patterns in dialysis patient care technician staffing. We found that US dialysis facilities varied considerably in their patient care technician staffing, and these variations were related to geography, time, and various facility characteristics. Future studies, including both observational and intervention studies, could use these data to generate hypotheses and target facilities for interventions, addressing some of the limitations in our study by collecting more detailed pre- and postpandemic staffing data; provider-level data on burnout, work experiences, and turnover intention; and facility- and/or patient-level data on quality of care and outcomes. Such work could lead to better, more stable dialysis patient care technician staffing, potentially relieving extra burdens on the entire dialysis workforce and improving dialysis care.
