# Supplementary material for: Patient Care Technician Staffing in US Hemodialysis Facilities: An Ecological Study
Source: Kidney Med. 2023 Dec 15;6(3):100782. doi: 10.1016/j.xkme.2023.100782 (PMC10900094; doi:10.1016/j.xkme.2023.100782)
Supplement: Supplementary File (PDF) — Figure S1-S3, Table S1-S5. [file mmc1.pdf]

Fig S1. Median U.S. dialysis facility HD station:patient care technician ratios in 2019, by state

Data from states with 10 or fewer facilities are suppressed.

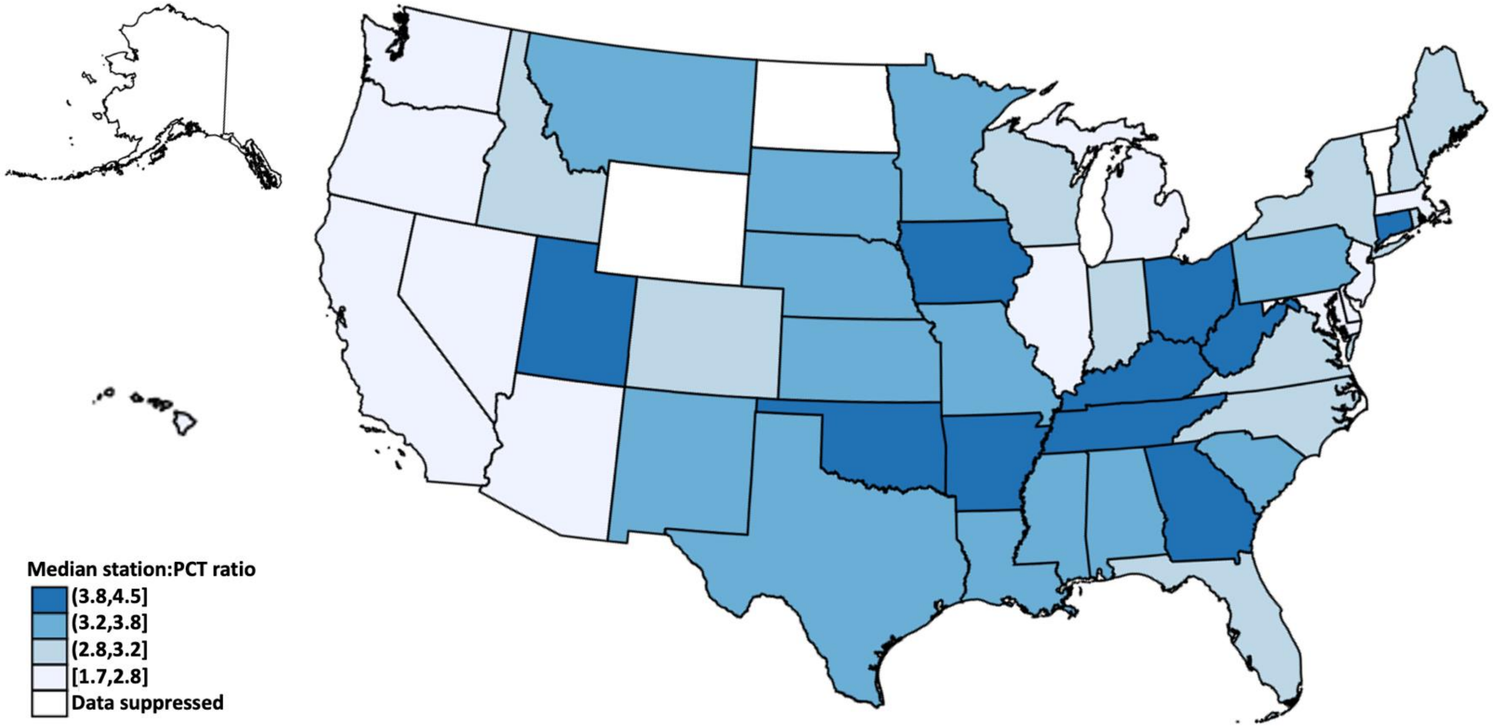

Fig S2. Median facility HD station:patient care technician ratios (bars) and mean percentage of dialysis patient care technician positions reported as open (squares) at U.S. dialysis facilities, by year

P<0.001 across year for both. PCT, patient care technician.

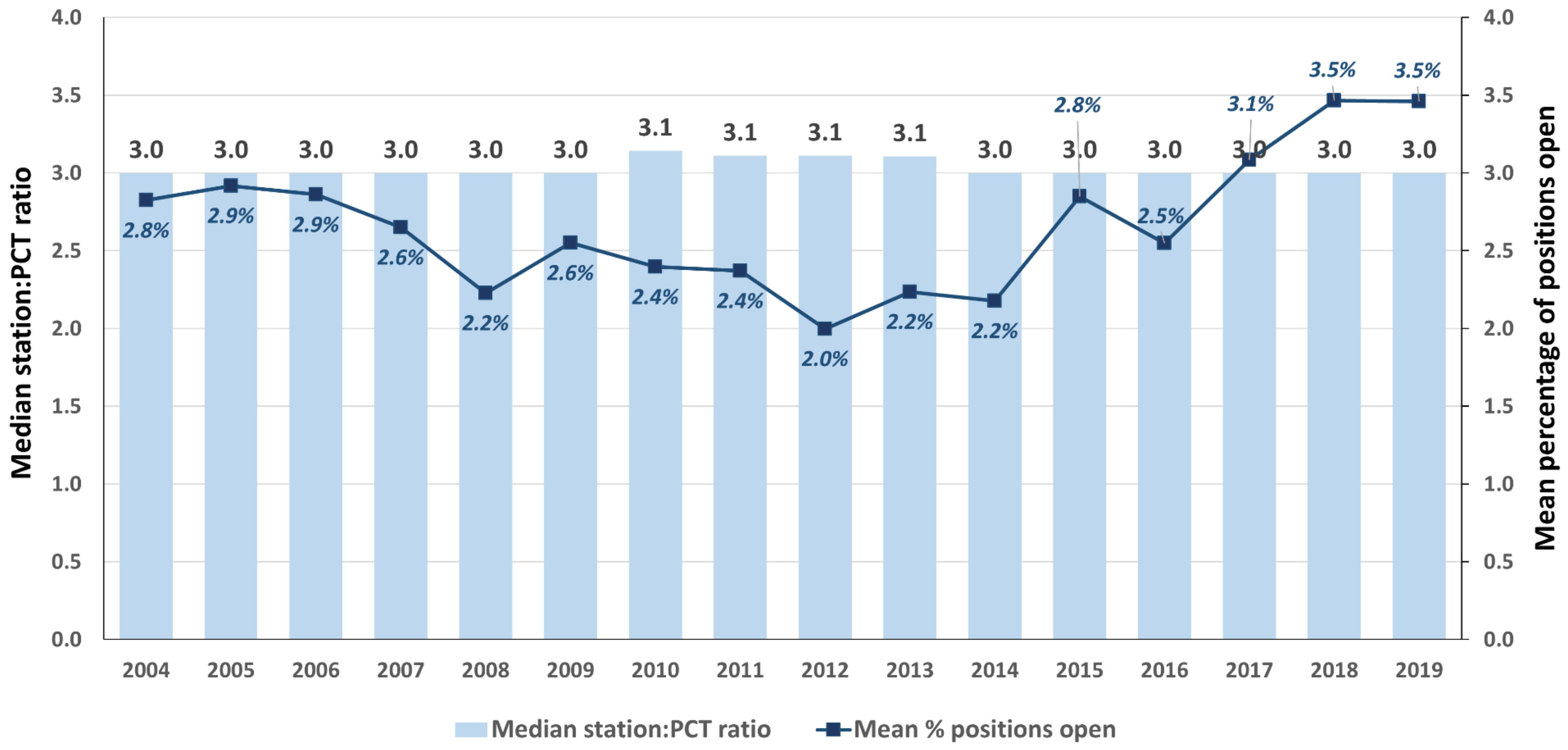

Fig S3. Scatter plots and correlations between patient:PCT ratios and selected continuous charateristics of facilities

A, number of hemodialysis stations; B, number of patients receiving hemodialysis; C, patient:registered nurse ratio; D, patient:social worker ratio; E, percentage of patients waitlisted; F, percentage of individuals living in poverty in facility zip code. Lines represent lowess smoothing.

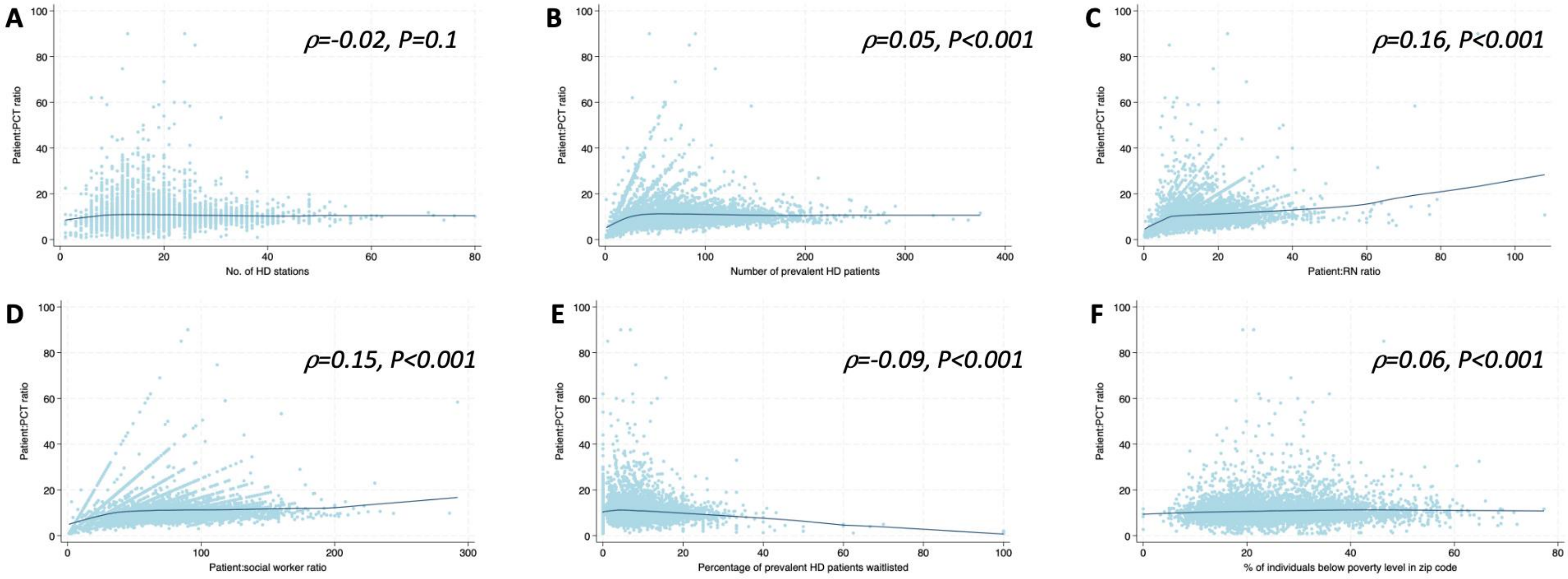

Table S1. Details of U.S. state dialysis staffing mandates

State	Ratio type(s), per shift	Ratio value
Georgia[a]	Patient:qualified dialysis care giver (at least 1 licensed RN on floor)	≤4:1
Maryland[b]	Patient:direct patient care providers	≤3:1
Massachusetts[c]	Patient:nursing staff (RNs or LVNs/LPNs trained in chronic dialysis)	≤3:1
New Jersey[d]	Patient:RN (inpatient)	≤3:1
Oregon[e]	Patient:licensed RN, LVN/LPN, or CHDT	≤4:1
South Carolina[f]	Patient:direct care staff	≤4:1
Texas[g]	Patient:direct care staff (at least 1 RN)	≤3.5:1
Utah[h]	Clinic staff personnel (at least 1 RN):patient<br>Patient:(PCT or LPN)	≥2:1<br>≤4:1

CHDT, certified hemodialysis technician; LVN, licensed vocational nurse; LPN, licensed practical nurse; PCT, patient care technician; RN, registered nurse.

[a]Rules and Regulations of the State of Georgia. Rule 111-8-22-.06(2)(b)4(i).

[b]Md. Code Regs. 10.30.02.04.D1.

[c]Massachusetts Department of Public Health, 150 CMR 145.510: M.G.L. c. 111, §§ 3, 51A and 53.

[d]N.J. Admin Code §8:43G-30.6.

[e]South Carolina Regulation 61-97§504B.

[f]Oregon Health Authority 333-700-0100.

[g]Texas Administrative Code Rule §-175.45.d. Staffing minimums vary based on total number of patients on shift: 1-7, 1 RN and 1 RN, LVN, or PCT required; 8 patients, 1 RN and 2 RN, LVN, or PCT; 9-12, 1 RN and 3 RN, LVN/PCT; 13-16 patients, 1 RN, 1 RN/LVN, and 4 RN/LVN/PCT; etc.

[h]Utah Admin. Code 432-650-7.

Table S2. Patient:PCT and HD station:PCT ratios in U.S. facilities as of 12/31/2019, by ESRD Network

Network[a]	N (%)	Median (IQR) patient:PCT ratio	Median (IQR) station:PCT ratio
1 (Connecticut, Maine, Massachusetts, New Hampshire, Rhode Island, Vermont)	190 (2.8%)	10.5 (8.6-13.3)	2.9 (2.2-4.0)
2 (New York)	299 (4.4%)	12.1 (9.9-15.3)	3.0 (2.0-4.8)
3 (New Jersey, Puerto Rico, U.S. Virgin Islands)	185 (2.7%)	10.5 (9.3-12.5)	2.7 (2.2-3.8)
4 (Pennsylvania)	306 (4.5%)	10.0 (8.4-12.0)	3.4 (2.6-4.8)
5 (Delaware, District of Columbia, Maryland, Virginia, West Virginia)	438 (6.4%)	8.6 (7.0-10.3)	2.7 (2.0-3.8)
6 (Georgia, North Carolina, South Carolina)	698 (10.2%)	10.3 (8.7-12.5)	3.5 (2.7-4.8)
7 (Florida)	451 (6.6%)	9.9 (8.4-12.0)	3.1 (2.3-4.3)
8 (Alabama, Mississippi, Tennessee)	441 (6.4%)	10.7 (8.8-13.4)	3.8 (2.9-5.0)
9 (Indiana, Kentucky, Ohio)	580 (8.5%)	10.0 (8.3-12.2)	3.7 (2.7-5.2)
10 (Illinois)	261 (3.8%)	9.2 (7.7-10.7)	2.6 (2.2-3.6)
11 (Michigan, Minnesota, North Dakota, South Dakota, Wisconsin)	451 (6.6%)	9.3 (7.8-11.7)	3.0 (2.3-4.0)
12 (Iowa, Kansas, Missouri, Nebraska)	314 (4.6%)	10.0 (8.3-12.5)	4.0 (2.9-5.3)
13 (Arkansas, Louisiana, Oklahoma)	323 (4.7%)	10.4 (8.7-14.0)	3.7 (2.7-5.1)
14 (Texas)	699 (10.2%)	10.5 (8.7-13.0)	3.3 (2.5-4.8)
15 (Arizona, Colorado, Nevada, New Mexico, Utah, Wyoming)	350 (5.1%)	9.3 (7.9-11.0)	2.7 (2.0-4.0)
16 (Alaska, Idaho, Montana, Oregon, Washington)	215 (3.1%)	8.0 (7.0-9.3)	2.0 (1.6-3.0)
17 [California (Northern[b]), Hawaii, American Samoa, Guam, Mariana Islands)	245 (3.6%)	8.8 (7.8-10.5)	2.1 (1.7-2.9)
18 [California (Southern[b])]	416 (6.1%)	9.6 (8.3-10.9)	2.0 (1.6-2.5)

IQR, interquartile range (25th, 75th percentile).

[a]P<0.001 across Networks by equality-of-medians test.

[b]Estimated via facility ZIP code.

Table S3. Sensitivity analysis: association of selected facility characteristics with 2019 U.S. facility HD station:patient care technician ratios

**Characteristic**	**Median (IQR) HD station:PCT ratio**	**Difference in HD station:PCT ratio [β (95% CI)[a]]**		
		**Crude**	**Model 1[b]**	**Model 2[b]**
Ownership				
For-profit	3.0 (2.3-4.3)	1.00 (ref.)	1.00 (ref.)	1.00 (ref.)
Not-for-profit	3.1 (2.1-4.9)	0.02 (-0.10,0.13)	0.17 (0.04,0.29)	0.18 (0.06,0.31)
P[c]	*0.4*			
Large dialysis organization				
Yes	3.2 (2.2-4.8)	1.00 (ref.)	1.00 (ref.)	1.00 (ref.)
No	3.0 (2.3-4.2)	0.06 (-0.02,0.14)	0.09 (0.01,0.17)	0.06 (-0.03,0.14)
P[c]	*0.07*			
Type				
Freestanding	3.0 (2.3-4.3)	1.00 (ref.)	1.00 (ref.)	1.00 (ref.)
Hospital-based	3.0 (2.0-5.5)	-0.16 (-0.34,0.02)	-0.59 (-0.77,-0.40)	-0.56 (-0.75,-0.37)
P[c]	*0.9*			
Number of stations				
< 18	3.3 (2.4-4.8)	1.00 (ref.)	1.00 (ref.)	1.00 (ref.)
≥ 18	2.9 (2.2-4.0)	-0.37 (-0.44,-0.30)	0.83 (0.76,0.91)	0.82 (0.75,0.90)
P[c]	*<0.001*			
Patient:registered nurse ratio				
≤ 15	3.3 (2.4-5.0)	1.00 (ref.)	1.00 (ref.)	1.00 (ref.)
> 15	2.9 (2.2-4.0)	-0.33 (-0.40,-0.26)	0.03 (-0.03,0.10)	0.00 (-0.07,0.06)
P[c]	*<0.001*			
Patient:social worker ratio				
≤ 70	4.0 (2.7-5.3)	1.00 (ref.)	1.00 (ref.)	1.00 (ref.)
> 70	2.6 (2.0-3.4)	-0.93 (-1.00,-0.87)	-0.34 (-0.41,-0.27)	-0.33 (-0.40,-0.26)
P[c]	*<0.001*			
Advanced practice provider present				
No	3.0 (2.3-4.3)	1.00 (ref.)	1.00 (ref.)	1.00 (ref.)
Yes	3.0 (2.2-4.3)	-0.04 (-0.21,0.13)	0.16 (0.01,0.31)	0.14 (-0.01,0.29)
P[c]	*0.7*			
LVN/LPN present				

Characteristic	Median (IQR) HD station:PCT ratio	Difference in HD station:PCT ratio [β (95% CI)[a]]		
		Crude	Model 1[b]	Model 2[b]
No	3.0 (2.3-4.3)	1.00 (ref.)	1.00 (ref.)	1.00 (ref.)
Yes	3.2 (2.2-4.8)	0.13 (-0.01,0.28)	0.42 (0.30,0.55)	0.40 (0.28,0.53)
P[c]	*0.1*			
Percentage of patients who are Black				
≤ 25%	3.0 (2.3-4.2)	1.00 (ref.)	1.00 (ref.)	1.00 (ref.)
> 25%	3.0 (2.2-4.4)	0.05 (-0.02,0.12)	0.12 (0.06,0.18)	-0.03 (-0.15,0.08)
P[c]	*0.5*			
Percentage of patients with permanent vascular access in place/maturing at dialysis start				
≤ 40%	3.1 (2.3-4.4)	1.00 (ref.)	1.00 (ref.)	1.00 (ref.)
> 40%	3.0 (2.3-4.3)	-0.09 (-0.16,-0.01)	-0.12 (-0.18,-0.06)	0.02 (-0.07,0.12)
P[c]	*0.004*			
Percentage of patients on kidney transplant waitlist				
≤ 7%	3.3 (2.5-4.7)	1.00 (ref.)	1.00 (ref.)	1.00 (ref.)
> 7%	2.8 (2.1-4.0)	-0.39 (-0.46,-0.32)	-0.14 (-0.20,-0.08)	-0.12 (-0.19,-0.06)
P[c]	*<0.001*			
Percentage of patients with functional impairment				
≤ 10%	3.0 (2.2-4.2)	1.00 (ref.)	1.00 (ref.)	1.00 (ref.)
> 10%	3.2 (2.3-4.5)	0.19 (0.12,0.26)	0.07 (0.01,0.13)	0.03 (-0.06,0.11)
P[c]	*<0.001*			
Facility in poverty area[d]				
No	3.0 (2.2-4.3)	1.00 (ref.)	1.00 (ref.)	1.00 (ref.)
Yes	3.0 (2.3-4.3)	0.06 (-0.02,0.13)	0.18 (0.11,0.24)	0.12 (0.05,0.19)
P[c]	*0.4*			

N=6815 (excluding observations with missing data on covariates). IQR, interquartile range (25th-75th percentiles); PCT, patient care technician.
[a]From robust regression.[10]
[b]Model 1 adjusted for facility characteristics: ownership (profit/not-for-profit), large dialysis organization (yes vs. no), number of prevalent in-center hemodialysis patients. Model 2 adjusted for variables Model 1 plus patient and neighborhood characteristics: percentage of patients who were aged ≥65, percentage of patients who were Black, percentage of patient with diabetes, percentage of patients with functional impairment, percentage of patients with a permanent vascular access in place at dialysis start, and percentage of patients with known pre-end-stage kidney disease care.
[c]By Wilcoxon rank sum test.
[d]*N*=6745. Poverty area defined as a ZIP code tabulation area with ≥20% of households living below the poverty level.

Table S4. Sensitivity analysis: association of selected facility characteristics with 2019 U.S. facility patient:patient care technician ratios among facilities with ≤10% change in in-center hemodialysis patient census over 5 years (2015-2019)

Characteristic	Median (IQR) HD patient:PCT ratio	Difference in HD patient:PCT ratio [β (95% CI)[a]]		
		Crude	Model 1[b]	Model 2[b]
Ownership				
For-profit	9.5 (7.5-11.7)	1.00 (ref.)	1.00 (ref.)	1.00 (ref.)
Not-for-profit	9.6 (7.5-14.2)	-0.20 (-0.75,0.35)	-0.01 (-0.74,0.71)	-0.09 (-0.81,0.64)
P[c]	*0.6*			
Large dialysis organization				
Yes	9.0 (7.0-12.2)	1.00 (ref.)	1.00 (ref.)	1.00 (ref.)
No	9.7 (7.8-11.7)	-0.80 (-1.23,-0.37)	-0.77 (-1.28,-0.26)	-0.75 (-1.26,-0.25)
P[c]	*0.09*			
Type				
Freestanding	9.5 (7.6-11.8)	1.00 (ref.)	1.00 (ref.)	1.00 (ref.)
Hospital-based	9.6 (6.9-15.4)	-0.84 (-1.49,-0.18)	-0.56 (-1.43,0.30)	0.02 (-0.85,0.89)
P[c]	*0.3*			
Number of stations				
< 18	9.3 (6.7-11.8)	1.00 (ref.)	1.00 (ref.)	1.00 (ref.)
≥ 18	9.8 (8.1-12.0)	0.89 (0.48,1.31)	-0.74 (-1.21,-0.27)	-0.83 (-1.30,-0.36)
P[c]	*<0.001*			
Patient:registered nurse ratio				
≤ 15	8.5 (6.0-11.2)	1.00 (ref.)	1.00 (ref.)	1.00 (ref.)
> 15	10.3 (8.7-12.5)	2.23 (1.83,2.62)	1.54 (1.11,1.96)	0.80 (0.65,0.94)
P[c]	*<0.001*			
Patient:social worker ratio				
≤ 70	8.3 (6.0-11.2)	1.00 (ref.)	1.00 (ref.)	1.00 (ref.)
> 70	10.3 (8.7-12.3)	2.32 (1.94,2.70)	1.42 (0.93,1.90)	1.48 (1.05,1.90)
P[c]	*<0.001*			
Advanced practice provider present				
No	9.6 (7.5-11.9)	1.00 (ref.)	1.00 (ref.)	1.00 (ref.)
Yes	9.5 (6.8-13.4)	0.16 (-0.74,1.06)	0.22 (-0.62,1.07)	0.30 (-0.54,1.14)
P[c]	*0.6*			
LVN/LPN present				

Characteristic	Median (IQR) HD patient:PCT ratio	Difference in HD patient:PCT ratio [β (95% CI)[a]]		
		Crude	Model 1[b]	Model 2[b]
No	9.5 (7.5-11.7)	1.00 (ref.)	1.00 (ref.)	1.00 (ref.)
Yes	12.2 (9.0-16.9)	2.08 (1.24,2.92)	1.56 (0.76-2.35)	1.49 (0.70,2.28)
P[c]	<0.001			
Percentage of patients who are Black				
≤ 25%	9.3 (7.3-11.7)	1.00 (ref.)	1.00 (ref.)	1.00 (ref.)
> 25%	9.8 (7.7-12.0)	0.53 (0.11,0.94)	0.42 (0.03,0.81)	0.51 (-0.24,1.25)
P[c]	*0.02*			
Percentage of patients with permanent vascular access in place/maturing at dialysis start				
≤ 40%	9.5 (7.0-11.8)	1.00 (ref.)	1.00 (ref.)	1.00 (ref.)
> 40%	9.7 (7.7-12.0)	0.36 (-0.06,0.78)	0.18 (-0.21,0.58)	0.19 (-0.42,0.80)
P[c]	*0.2*			
Percentage of patients on kidney transplant waitlist				
≤ 7%	9.6 (7.3-12.2)	1.00 (ref.)	1.00 (ref.)	1.00 (ref.)
> 7%	9.5 (7.6-11.7)	0.15 (-0.26,0.57)	-0.22 (-0.61,0.18)	-0.13 (-0.52,0.27)
P[c]	*>0.9*			
Percentage of patients with functional impairment				
≤ 10%	9.5 (7.4-11.7)	1.00 (ref.)	1.00 (ref.)	1.00 (ref.)
> 10%	9.6 (7.6-12.1)	0.24 (-0.18,0.66)	0.37 (-0.02,0.76)	0.67 (0.13,1.22)
P[c]	*0.2*			
Facility in poverty area[d]				
No	9.2 (7.3-11.2)	1.00 (ref.)	1.00 (ref.)	1.00 (ref.)
Yes	9.8 (7.7-12.2)	0.52 (0.08,0.97)	0.29 (-0.13,0.71)	0.38 (-0.04,0.80)
P[c]	*0.002*			

N=1158 (excluding observations with missing data on covariates). IQR, interquartile range (25th-75th percentiles); PCT, patient care technician.
[a]From robust regression.[10]
[b]Model 1 adjusted for facility characteristics: ownership (profit/not-for-profit), large dialysis organization (yes vs. no), number of prevalent in-center hemodialysis patients. Model 2 adjusted for variables Model 1 plus patient and neighborhood characteristics: percentage of patients who were aged ≥65, percentage of patients who were Black, percentage of patient with diabetes, percentage of patients with functional impairment, percentage of patients with a permanent vascular access in place at dialysis start, and percentage of patients with known pre-end-stage kidney disease care.
[c]By Wilcoxon rank sum test.
[d]*N*=1137. Poverty area defined as a ZIP code tabulation area with ≥20% of households living below the poverty level.

Table S5. Sensitivity analysis: association of selected facility characteristics with 2019 U.S. facility HD patient:patient care technician ratios among facilities with ≤5% change in in-center hemodialysis patient census over 5 years (2015-2019)

Characteristic	Median (IQR) HD patient:PCT ratio	Difference in HD patient:PCT ratio [β (95% CI)[a]]		
		Crude	Model 1[b]	Model 2[b]
Ownership				
For-profit	7.8 (5.5-10.2)	1.00 (ref.)	1.00 (ref.)	1.00 (ref.)
Not-for-profit	9.1 (7.1-12.6)	0.98 (0.07,1.88)	0.78 (-0.38,1.94)	0.64 (-0.50,1.78)
P[c]	*<0.001*			
Large dialysis organization				
Yes	7.7 (5.6-10.9)	1.00 (ref.)	1.00 (ref.)	1.00 (ref.)
No	8.5 (6.0-10.5)	-0.66 (-1.38,0.06)	-0.66 (-1.40,0.08)	-0.81 (-1.53,-0.07)
P[c]	*0.5*			
Type				
Freestanding	8.0 (5.6-10.3)	1.00 (ref.)	1.00 (ref.)	1.00 (ref.)
Hospital-based	9.0 (6.7-12.6)	0.58 (-0.42,1.59)	-0.36 (-1.64,0.91)	0.16 (-1.11,1.43)
P[c]	*0.01*			
Number of stations				
< 18	7.5 (5.3-10.1)	1.00 (ref.)	1.00 (ref.)	1.00 (ref.)
≥ 18	9.0 (6.5-10.9)	1.17 (0.47-1.86)	-0.82 (-1.52,-0.13)	-0.81 (-1.49,-0.12)
P[c]	*0.002*			
Patient:registered nurse ratio				
≤ 15	6.3 (4.0-8.7)	1.00 (ref.)	1.00 (ref.)	1.00 (ref.)
> 15	9.7 (7.7-11.9)	3.47 (2.87,7.07)	2.43 (1.76,3.10)	2.32 (1.66,2.97)
P[c]	*<0.001*			
Patient:social worker ratio				
≤ 70	6.0 (4.0-8.0)	1.00 (ref.)	1.00 (ref.)	1.00 (ref.)
> 70	9.8 (8.1-12.2)	3.86 (3.29,4.44)	2.94 (2.16,3.72)	2.85 (2.08,3.62)
P[c]	*<0.001*			
Advanced practice provider present				
No	8.0 (6.0-10.5)	1.00 (ref.)	1.00 (ref.)	1.00 (ref.)
Yes	8.5 (5.0-11.0)	-0.26 (-1.72,1.20)	-0.36 (-1.60,0.87)	-0.42 (-1.63,0.78)
P[c]	0.8			
LVN/LPN present				

Characteristic	Median (IQR) HD patient:PCT ratio	Difference in HD patient:PCT ratio [β (95% CI)[a]]		
		Crude	Model 1[b]	Model 2[b]
No	8.0 (5.8-10.5)	1.00 (ref.)	1.00 (ref.)	1.00 (ref.)
Yes	11.8 (8.3-19.3)	3.07 (1.41,4.73)	0.99 (-0.43,2.41)	0.84 (-0.55,2.23)
P[c]	*<0.001*			
Percentage of patients who are Black				
≤ 25%	8.0 (6.0-10.5)	1.00 (ref.)	1.00 (ref.)	1.00 (ref.)
> 25%	8.3 (6.0-10.9)	0.17 (-0.55,0.88)	0.08 (-0.52,0.68)	0.31 (-0.85,1.46)
P[c]	*0.6*			
Percentage of patients with permanent vascular access in place/maturing at dialysis start				
≤ 40%	8.0 (5.6-10.5)	1.00 (ref.)	1.00 (ref.)	1.00 (ref.)
> 40%	8.0 (6.0-10.8)	0.17 (-0.53,0.88)	-0.17 (-0.76,0.43)	-0.05 (-0.95,0.85)
P[c]	*0.6*			
Percentage of patients on kidney transplant waitlist				
≤ 7%	7.7 (5.3-10.6)	1.00 (ref.)	1.00 (ref.)	1.00 (ref.)
> 7%	8.7 (6.3-10.7)	0.75 (0.05-1.45)	0.04 (-0.56.0.64)	0.12 (-0.48,0.72)
P[c]	0.08			
Percentage of patients with functional impairment				
≤ 10%	8.5 (5.5-10.7)	1.00 (ref.)	1.00 (ref.)	1.00 (ref.)
> 10%	8.0 (6.0-10.5)	0.20 (-0.51,0.91)	0.59 (0.00,1.18)	0.96 (0.12,1.79)
P[c]	*0.7*			
Facility in poverty area[d]				
No	7.5 (5.5-9.4)	1.00 (ref.)	1.00 (ref.)	1.00 (ref.)
Yes	8.5 (6.0-11.3)	0.81 (0.05,1.57)	0.28 (-0.36,0.92)	0.38 (-0.27,1.02)
P[c]	0.003			

N=477 (excluding observations with missing data on covariates). IQR, interquartile range (25th-75th percentiles); PCT, patient care technician.
[a]From robust regression.
[b]Model 1 adjusted for facility characteristics: ownership (profit/not-for-profit), large dialysis organization (yes vs. no), number of prevalent in-center hemodialysis patients. Model 2 adjusted for variables Model 1 plus patient and neighborhood characteristics: percentage of patients who were aged ≥65, percentage of patients who were Black, percentage of patient with diabetes, percentage of patients with functional impairment, percentage of patients with a permanent vascular access in place at dialysis start, and percentage of patients with known pre-end-stage kidney disease care.
[c]By Wilcoxon rank sum test.
[d]*N*=471. Poverty area defined as a ZIP code tabulation area with ≥20% of households living below the poverty level.
